# Acute Toxic Effects and Toxicity-Source Analysis of CuO, ZnO, and SiO_2_ Nanoparticles in River–Lake Water Bodies

**DOI:** 10.3390/biology15212020

**Published:** 2026-11-06

**Authors:** Jing Hua, Xiaowei Xu, Jie Gu, Yufeng Xie, Anfei He, Qi Yu, Lei Wang, Chang Liu

**Affiliations:** 1 Nanjing Institute of Environmental Sciences, Ministry of Ecology and Environment of China, Nanjing 210042, China; huajing@nies.org (J.H.);; 2 State Key Laboratory of Pollution Control and Resource Reuse, College of Environmental Science and Engineering, Tongji University, Shanghai 200092, China; 3 School of Environmental Science and Engineering, Suzhou University of Science and Technology, Suzhou 215009, China

**Keywords:** nano-metal oxides, *Vibrio fischeri*, bioluminescence inhibition assay, dissolution, toxicity-source analysis, hormesis, river–lake water

## Abstract

Engineered nano-metal oxides discharged into river–lake systems interact with water chemistry before reaching biota, yet the fraction of their acute toxicity that derives from the particles themselves, rather than from dissolved ions, remains unresolved in authentic natural waters. This study determined the acute toxic effects of CuO, ZnO, and SiO_2_ nanoparticles at a concentration of 100 mg/L dispersed in water samples from Taihu Lake and Suzhou River (two representative and polluted water bodies in eastern China) on *Vibrio fischeri* (*V. fischeri*), and partitioned each measured inhibition E(total) into an ion-attributed component E(ion) and a particle-attributed component E(particle) using paired assays of the whole suspension and of its 0.02 µm filtrate. Aggregation, surface charge, and dissolution kinetics were followed for 72 h (15 d for SiO_2_). CuO nanoparticles aggregated moderately in both natural waters and released at most 0.087 mg/L Cu^2+^, so that the persistent suspension inhibition of 0.09–0.64% coexisted with a mildly stimulatory ion fraction (−0.19% to 0.01%), leaving the particle component to account for 81.1–94.0% of the measured effect. ZnO nanoparticles released up to 7.15 mg/L Zn^2+^ in Suzhou River water, where inhibition rose in step with the dissolved fraction and the ion component contributed 56.8–95.6% of the effect; in Taihu Lake water, both the suspension and the ion fraction remained stimulatory. SiO_2_ nanoparticles and the released reactive silicate induced no acute inhibition over 15 d (mean −0.024% and −0.013% in the two waters, both significantly below zero) despite the net dissolution solubility reaching up to 32.4% of the nominal dose. The toxicity source therefore differs by material within the same water matrix, indicating that risk-management measures for CuO should target particle transport and settling, whereas measures for ZnO should suppress dissolution.

## Simple Summary

Tiny engineered particles known as nanoparticles are increasingly released into rivers and lakes through wastewater and industrial discharge, yet it remains unclear whether their harm to aquatic life comes from the particles themselves or from the metal ions they release as they dissolve. This study examined three common types of nanoparticles—copper oxide (CuO), zinc oxide (ZnO), and silicon dioxide (SiO_2_)—suspended in water collected from Taihu Lake and the Suzhou River in eastern China, using a light-emitting bacterium as a rapid toxicity sensor. By measuring both the whole particle mixture and its dissolved fraction at each time point, the toxicity of each component was separated. The results showed that CuO nanoparticles harmed the bacteria mainly through direct particle contact, while ZnO exerted its effect almost entirely through dissolved zinc ions, and SiO_2_ caused no acute harm despite substantial dissolution. These findings demonstrate that the dominant toxicity source differs by material, meaning that environmental protection measures should be tailored accordingly: controlling particle transport for CuO, but suppressing ion release for ZnO. This material-specific guidance can help regulators and water managers prioritize the most effective pollution-control strategy for each type of nanoparticle entering natural waters.

## 1. Introduction

Engineered nanoparticles reach river and lake systems through municipal and industrial wastewater discharge, stormwater runoff, and accidental releases [1,2], and monitoring campaigns in Chinese surface waters have begun to quantify the resulting exposure. Titanium-bearing nanoparticles were identified and sized in the water column of Lake Taihu using single-particle techniques [3], and a recent survey resolved the speciation, distribution, and environmental risk of the dominant silver-containing nanoparticles in the same lake [4]. Pollution incidents involving nanomaterials differ from these continuous low-dose emissions because pulse inputs can transiently raise local concentrations toward the mg/L range, so that hazard characterization for such scenarios relies on experiments at doses that represent episodic maxima rather than annual averages [5,6]. Whatever the release route, the physical, chemical, and biological transformations that engineered nanomaterials undergo once suspended in natural water—aggregation, sedimentation, surface-charge modification, and dissolution—determine which forms the organisms actually encounter [7], and reviews of ecological risk assessment now stress that these transformed forms, rather than the pristine particles, should anchor the assessment [2].

Among metal-oxide nanoparticles, CuO, ZnO, and SiO_2_ span nearly the full spectrum of expected aqueous behaviors, which makes their parallel examination in identical water matrices particularly informative [8,9,10]. CuO nanoparticles release Cu^2+^ slowly and persist as particles in natural aqueous matrices [11], although dissolved organic matter can accelerate their dissolution through copper complexation in simplified media [12]. ZnO nanoparticles dissolve readily, and their equilibrium and kinetic dissolution in surface waters responds strongly to pH and ligand availability [13]. SiO_2_ nanoparticles are the least soluble of the three, yet their aggregation behavior is highly responsive to the electrolyte composition of the receiving water [14]. A three-material comparison therefore interrogates the full range from particle-dominated to dissolution-dominated exposure within the same experimental frame.

Evidence on which fraction of a suspension actually causes the acute effect remains split between materials. Comparative experiments in which CuO nanoparticles and dissolved copper were dosed separately have repeatedly shown that the particles exert toxicity distinct from, and in addition to, the ions—in the sediment-dwelling oligochaete *Tubifex tubifex* (*T. tubifex*) [15], in *Drosophila melanogaster* (*D. melanogaster*) across bioaccumulation, survival, behavior, and fecundity endpoints [16], and in freshwater mesocosms where biotic and abiotic interactions governed particle fate and toxicity [17]. For ZnO nanoparticles the opposite pattern dominates the literature: their toxicity toward *V. fischeri* has been attributed specifically to dissolved Zn^2+^ [18], rotifer assays distinguish the ion-driven component of the response [19], and microalgal mixture studies explicitly treat the dissolved ions as the operative toxic species [20]. Silica nanoparticles, in contrast, show weak or no acute effects in most aquatic test systems, as summarized in a systematic review and meta-analysis [21] and an updated organism-level review [22]. Direct partitioning of a measured whole-suspension effect into ion- and particle-attributed components has nevertheless rarely been performed in authentic river–lake waters, and this is the gap the present study addresses.

The bioluminescent bacterium *V. fischeri* provides the acute endpoint for this partitioning because its 30 min luminescence assay is standardized, inexpensive, and sensitive to both dissolved and particulate toxicants. This luminescence inhibition assay follows international standard protocols (ISO 11348-3; OECD Test Guideline 249) [23], delivering rapid quantitative acute toxicity read-outs and enabling direct toxicity-source partitioning between particle-bound and dissolved-ion fractions under environmentally relevant natural-water matrices, and because it has been adopted repeatedly for nanomaterial screening, including for SiO_2_, cerium dioxide (CeO_2_), and aluminum oxide (Al_2_O_3_) abrasive particles [24], titanium dioxide (TiO_2_)-based photocatalysts [25], and silver–chitosan nanocomposites [26]. Two additional properties of this assay matter for interpretation. First, hormesis—stimulation of the test organism at low exposure levels—is a recognized complication of luminescent-bacterial testing and must be reported and interpreted rather than discarded as noise [27]. Second, the signal itself rests on well-characterized quorum-sensing and luciferase machinery whose regulation has been dissected genetically [28], which supports the use of luminescence as a consistent readout of bacterial fitness under chemical stress.

Despite increasing research attention toward metal-oxide nanoparticle aquatic hazards, most available toxicity data are obtained from simplified synthetic culture media. There remains a scarcity of quantitative experimental evidence that disentangles particle-specific toxicity from dissolved-ion toxicity under chemically complex real-world freshwater conditions, and the relative contribution of particle-driven effects is poorly constrained across different nanoparticle types. Accordingly, the present work was designed with four specific objectives: (1) to quantify the acute luminescence-inhibitory effects of CuO, ZnO and SiO_2_ nanoparticles toward *V. fischeri* in authentic Taihu Lake and Suzhou River water; (2) to partition the total measured acute toxicity into dissolved-ion-attributed and particle-attributed fractions via paired suspension–filtrate assays; (3) to compare the magnitude and dominant pattern of particle-specific toxicity among the three tested nanomaterials; and (4) to derive mechanistic insights and practical risk-management implications for metal-oxide nanoparticles in natural aquatic environments.

This study determined the acute toxic effects of copper oxide, zinc oxide, and silicon dioxide nanoparticles at a concentration of 100 mg/L dispersed in water samples from Taihu Lake and Suzhou River (two representative and polluted water bodies in eastern China) on *V. fischeri*. Aggregation and surface charge were characterized at the beginning of the exposure, dissolution was tracked over the full test period, and each inhibition reading was decomposed into an ion-attributed component and a particle-attributed component using paired assays of the whole suspension and of its filtrate. Because both components were measured rather than assumed, the resulting attribution identifies, for each material, the fraction of the acute effect that would respond to particle-focused measures (for example, settling or removal of suspended solids) as opposed to dissolution-focused measures (for example, coatings that suppress ion release).

## 2. Materials and Methods

### 2.1. Nanoparticles and Reagents

CuO nanoparticles (purity > 99%) and ZnO nanoparticles (purity > 97%) were purchased from Sigma-Aldrich (St. Louis, MO, USA), and SiO_2_ nanoparticles (purity 99.5%) from Aladdin Reagent Co., Ltd. (Shanghai, China). Transmission electron microscopy (TEM, H-600, Hitachi, Japan) observations of the three types of nanoparticles were basically consistent with the information provided by the supplier. None of the three nanoparticles had surface coatings, and the differences in their purity came from the different preparation methods. To directly study the migration and transformation behavior of commercial nanoparticles in the environment, the purchased nanoparticles were used directly in subsequent experiments without any purification or surface modification (Table 1).

Suwannee River natural organic matter (NOM, catalog 2R101N) was obtained from the International Humic Substances Society (St. Paul, MN, USA). Lyophilized *V. fischeri* powder was purchased from Beijing Hamamatsu Photon Techniques Inc. (Beijing, China). Hydrochloric acid, ammonium molybdate, oxalic acid, and the other analytical-grade reagents were obtained from Sinopharm Chemical Reagent Co., Ltd. (Shanghai, China), and the SiO_2_ standard stock solution (100 mg/L) from the National Research Center for Certified Reference Materials of China (Beijing, China).

### 2.2. Water Sampling and Characterization

Surface water was collected at Li Lake Park in Wuxi (Taihu Lake) and at the Changfeng Park reach of the Suzhou River in Shanghai. Both samples were filtered through 0.45 µm cellulose acetate membranes on the day of collection and stored in the dark at 4 °C until use. Water temperature was recorded in situ, and the concentrations of the major cations and anions, total organic carbon, conductivity, and pH were determined in the laboratory; ionic strength was calculated from the major-ion composition. The two waters differ mainly in conductivity, nitrate, and organic-carbon content (Table 2), and neither contained detectable dissolved Cu^2+^ or Zn^2+^.

### 2.3. Nanoparticle Suspensions

Nanoparticle suspensions were prepared at 100 mg/L in six matrices: ultrapure water (UPW), an inorganic salt solution, an inorganic salt solution with NOM, a NOM solution (the four simulated matrices), Taihu Lake water, and Suzhou River water. The dose of 100 mg/L was selected to represent the upper concentration range that pulse releases of nanomaterials could reach in a receiving water body and that is therefore relevant for incident-driven ecological risk assessment. Each suspension was dispersed by ultrasonication using a KQ5200DE digital ultrasonic cleaner (Kunshan Ultrasonic Instruments Co., Ltd., Kunshan, Jiangsu, China) at a nominal power of 150 W and frequency of 38 kHz for 20 min at a water-bath temperature of 25 ± 2 °C and used immediately. The Z-average hydrodynamic diameter and the polydispersity index were measured by dynamic light scattering, and the zeta potential and pH were recorded at 0.5 h and 6 h after preparation.

### 2.4. Dissolution Experiments

To follow metal-ion release, the CuO and ZnO suspensions were stirred continuously (25 °C, 250 rpm) and 15 mL aliquots were withdrawn at 0, 1, 3, 9, 24, 48, and 72 h. Each aliquot was centrifuged at 20,000× *g* for 40 min at 25 °C, and the supernatant was passed through 0.22 µm water filter heads and then 0.02 µm cellulose acetate membrane filters. This sequential centrifugation-and-filtration protocol was adopted to ensure progressive removal of particles down to the 0.02 µm cut-off, which is smaller than the primary particle sizes of all three nanoparticles and well below their aggregated hydrodynamic diameters in the natural waters. The filtrates were acidified with concentrated nitric acid (1:50, *v*/*v*) and analyzed for Cu^2+^ and Zn^2+^ by inductively coupled plasma optical emission spectrometry (Agilent 720ES; Agilent Technologies Inc., Santa Clara, CA, USA). Three replicate filtrates were prepared at each time point.

For SiO_2_, the suspensions were stirred under the same conditions for 15 d and sampled daily. After ultrafiltration (4000× *g*, 30 min), the reactive silicate concentration in the filtrate was determined by the silicomolybdenum blue method (SL 91.1-1994) with the reagents listed in Table 3. In brief, at pH 1.2 ammonium molybdate reacts with silicic acid to form the yellow silicomolybdic heteropoly acid, which is reduced to silicomolybdenum blue by 1,2,4-aminonaphtholsulfonic acid; the absorbance was read at 660 nm on a UV–visible spectrophotometer (Evolution 201; Thermo Fisher Scientific Inc., Waltham, MA, USA) and converted to concentration using a standard curve. Three replicate filtrates were prepared at each time point. Pseudo-first-order rate constants were fitted to the rising phase of each concentration series by nonlinear least squares.

### 2.5. Vibrio fischeri Culture and the Acute Toxicity Assay

The bioluminescent marine bacterium *V. fischeri* (strain MCCC 1H00020), obtained from the Marine Culture Collection of China (MCCC, Xiamen, China), was used as the test organism. Lyophilized *V. fischeri* was revived with the resuscitation solution supplied by the manufacturer, streaked on the solid medium described in Table 4, and incubated in the dark at 20 ± 2 °C for 24 h. Single colonies were transferred to the liquid medium and grown with shaking (180 rpm) in the dark at 20 ± 2 °C; the culture was passaged on solid medium every two days to preserve activity. Based on the recorded growth curve, cells from the 14 h liquid culture, at which point the luminescence signal is stable, were used for all toxicity tests. This assay configuration follows the common practice for metal-oxide nanoparticle testing with bioluminescent bacteria [29].

At each designated exposure time (0, 1, 3, 9, 24, 48, and 72 h for CuO and ZnO; days 1–15 for SiO_2_), two aliquots were withdrawn simultaneously from the same stirred suspension described in Section 2.4. One aliquot was processed for dissolved-metal analysis, and the other (5 mL) was used for the luminescence assay, so that the dissolution data and the toxicity data correspond to identical samples. To this aliquot, 0.15 g of NaCl was added to maintain osmotic conditions and to prevent the salinity of the natural waters from interfering with bacterial luminescence. Assays were carried out in 96-well plates with three replicate wells per treatment, together with the 3% NaCl control solution (D1) and the corresponding sample (D2). After 30 min of static incubation in the dark, the luminescence intensity was recorded with a GloMax multi-detection system (Promega Corporation, Madison, WI, USA). The system provides a linear dynamic range of 9 orders of magnitude, ensuring that all luminescence measurements fell within the linear response range. The inhibition *E* was calculated according to Equation (1), where positive values denote inhibition of luminescence (toxicity) and negative values denote stimulation (hormesis):

(1) E = (D1 − D2)/D1 × 100%

The toxicity source was then partitioned according to Equation (2), where E(total) is the inhibition measured for the whole nanoparticle suspension, E(ion) is the inhibition measured for its 0.02 µm filtrate (the dissolved species), and E(particle) is the particle-attributed component obtained by difference:

(2) E(total) = 1 − (1 − E(ion))(1 − E(particle))

The ion and particle shares were then calculated as the marginal-contribution proportions according to Equations (3) and (4), where the ion share is the relative contribution of the dissolved species to the total effect, the particle share is the relative contribution of the particle-attributed component, and both are normalized to their sum:

(3) Ion share = |E(ion)(1 − E(particle))| / [|E(ion)(1 − E(particle))| + |E(particle)(1 − E(ion))|]

(4) Particle share = |E(particle)(1 − E(ion))| / [|E(ion)(1 − E(particle))| + |E(particle)(1 − E(ion))|]

### 2.6. Statistical Analysis

All experiments were performed in triplicate and results are reported as mean ± standard deviation. Paired *t*-tests were applied to compare E(total) and E(ion) time-series for each nanoparticle–water system. Associations between inhibition and dissolved-ion concentration were quantified with Pearson and Spearman correlation coefficients computed over the time-series of each system. One-sample *t*-tests were used for the 15 d SiO_2_ time-series to test whether inhibition values differed significantly from zero, evaluating potential hormesis. To control for type-I error inflation arising from multiple statistical tests, the Benjamini–Hochberg false discovery rate (FDR) correction was applied across the complete set of *t*-tests and correlation tests. Raw *p*-values were adjusted, and statistical significance was accepted at adjusted α = 0.05. For dissolution-kinetics post hoc pairwise comparisons, Benjamini–Hochberg FDR correction was also implemented for multiple group comparisons. All computations were performed in Python 3.14 using the NumPy and SciPy libraries.

## 3. Results

### 3.1. Aggregation and Surface Charge in the Six Matrices

**Figure 1:**
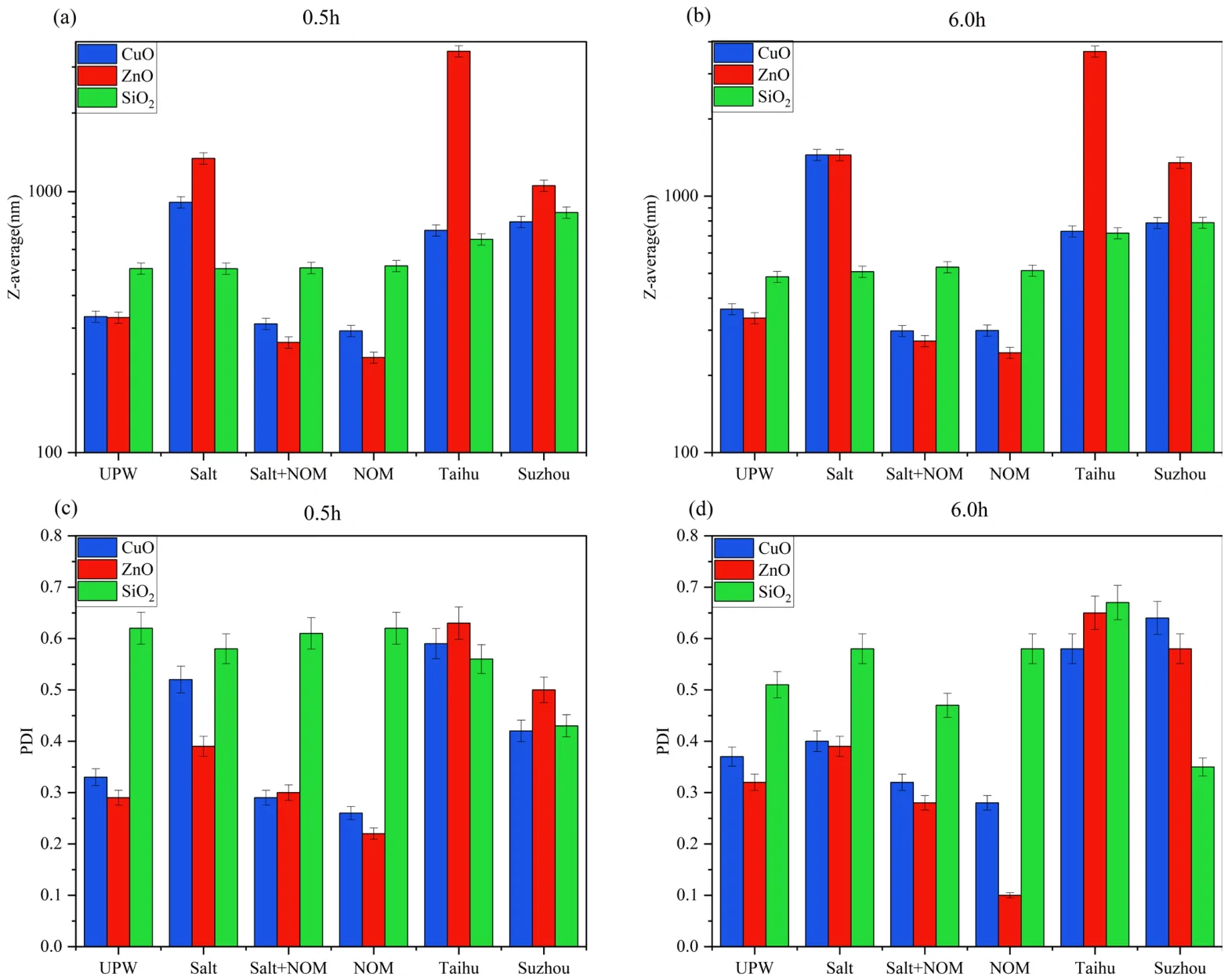
Hydrodynamic diameter and polydispersity index (PDI) of CuO, ZnO, and SiO_2_ nanoparticles (100 mg/L) in the six test solutions at 0.5 h and 6 h. (**a**,**b**) Z-average hydrodynamic diameter; (**c**,**d**) PDI. Solution codes: UPW, ultrapure water; Salt, inorganic salt solution; Salt + NOM, inorganic salt solution with natural organic matter; NOM, natural organic matter solution; Taihu, Taihu Lake water; Suzhou, Suzhou River water. Error bars show the standard deviation of triplicate dynamic light scattering runs.

The three nanoparticles behaved distinctly in the six matrices. CuO nanoparticles carried a positive zeta potential (+17.2 mV at 0.5 h) in UPW but reversed to negative values in every other matrix, reaching −12.2 mV in the inorganic salt solution and −18.1 mV in Taihu Lake water (6 h), consistent with the adsorption of anions and NOM onto the surface. Their hydrodynamic diameter nevertheless remained moderate in the natural waters, growing from roughly 710 nm at 0.5 h to 729 nm at 6 h in Taihu Lake water and from 765 to 786 nm in Suzhou River water, about twice the size measured in UPW (332–362 nm) and well below the 1.45 µm reached at 6 h in the inorganic salt solution (Figure 1a,b). The polydispersity indices at 6 h stayed at or below 0.07 in all matrices, indicating narrow aggregate populations (Figure 1c,d).

**Figure 2:**
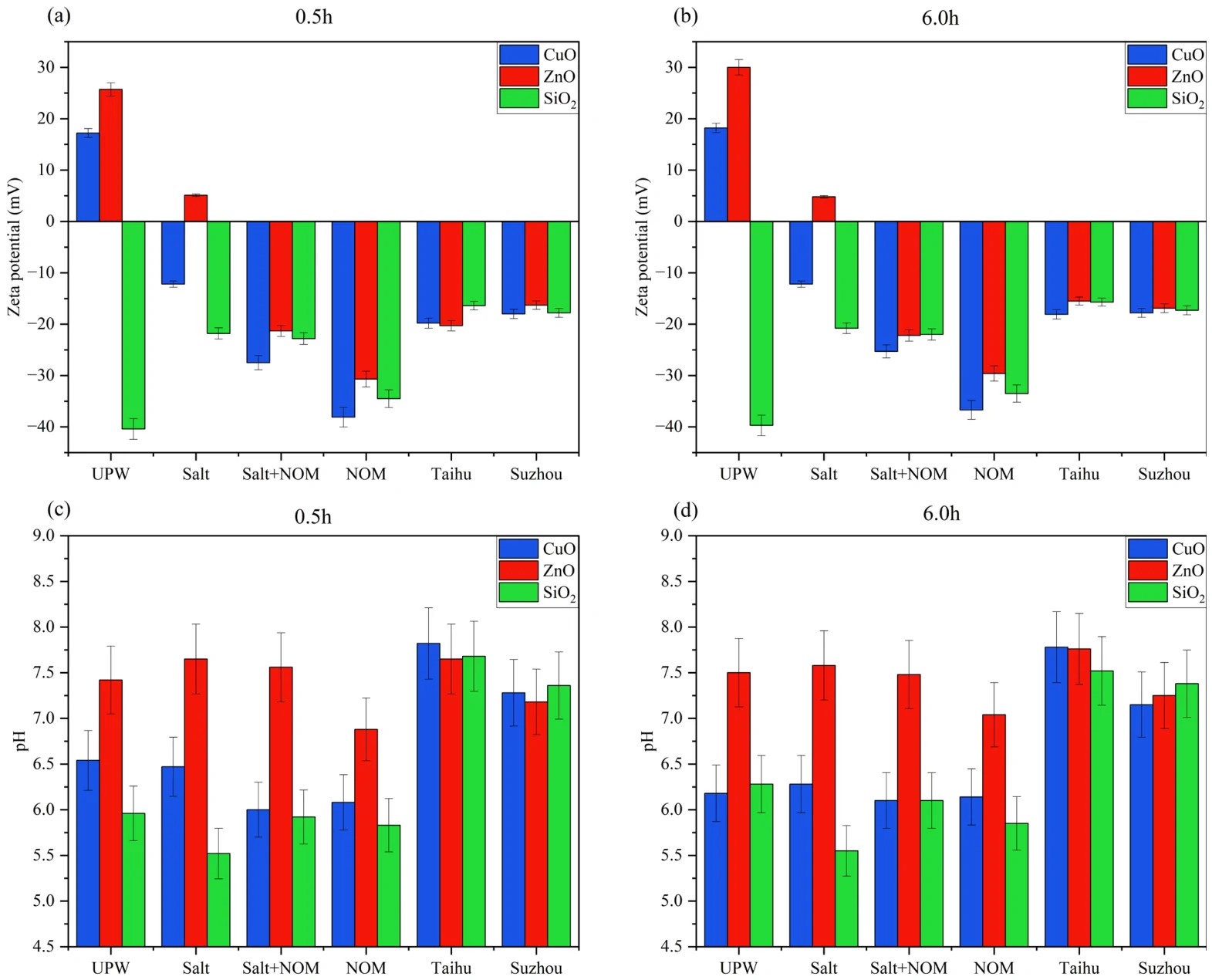
Zeta potential and pH of the three nanoparticle suspensions (100 mg/L) in the six test solutions at 0.5 h and 6 h. (**a**,**b**) Zeta potential, with the zero line drawn for reference; (**c**,**d**) pH. Solution codes as in Figure 1. Error bars show the standard deviation of triplicate measurements.

ZnO nanoparticles aggregated far more strongly in the natural waters: the Z-average reached 3.4–3.7 µm in Taihu Lake water, an order of magnitude above the ultrapure-water value, and 1.05–1.35 µm in Suzhou River water (Figure 1a,b). Their zeta potential was negative in both natural waters (−20.3 to −15.5 mV), and the pH of all ZnO suspensions remained between 7.0 and 7.8 (Figure 2c,d). SiO_2_ nanoparticles aggregated only mildly, from 0.48–0.51 µm in UPW to 0.66–0.83 µm in the natural waters, while their zeta potential rose from about −40 mV in UPW to −16 to −18 mV in the natural waters as counterions compressed the diffuse layer (Figure 2a,b). The aggregation state at 6 h is used below as the exposure-relevant particle size for the toxicity-source analysis, because it is the state the bacteria actually encounter during the 30 min assay.

### 3.2. Dissolution Kinetics

**Figure 3:**
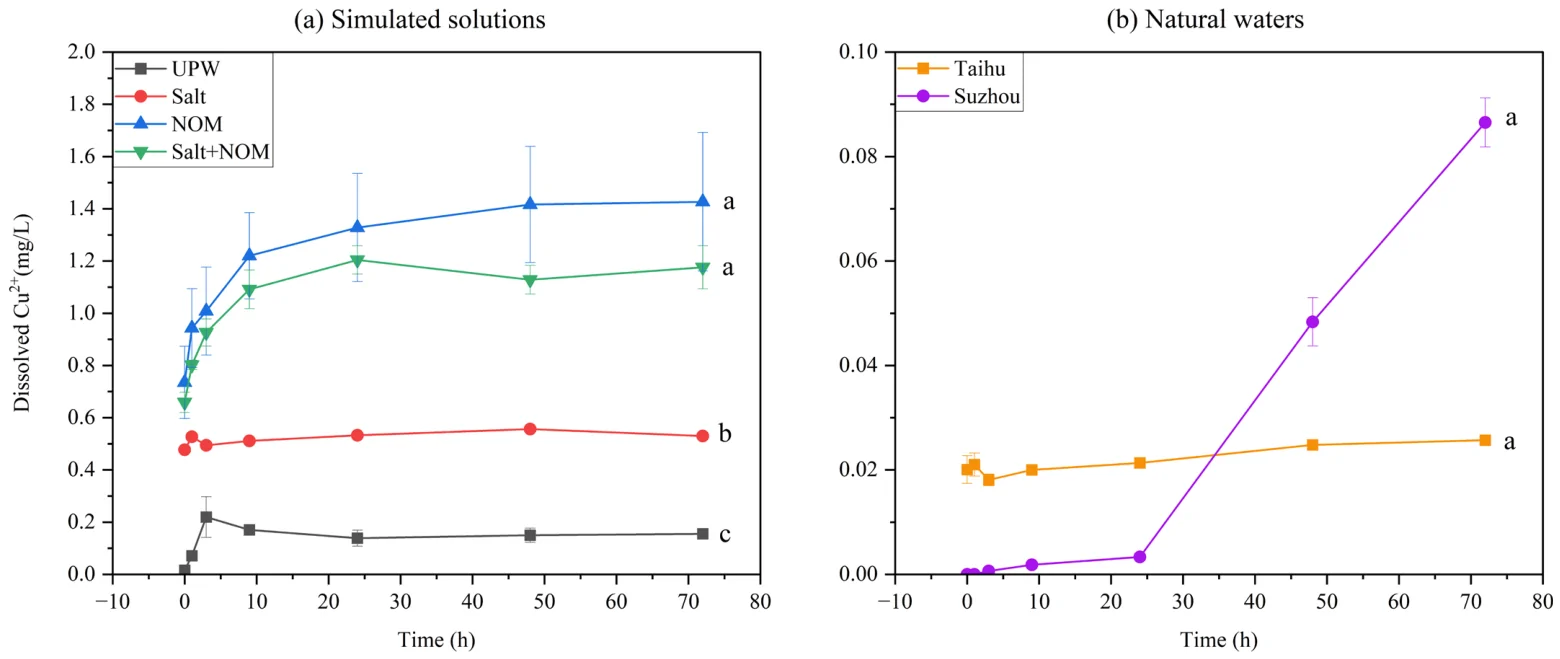
Dissolved Cu^2+^ released from CuO nanoparticles (100 mg/L) over 72 h in (**a**) the four simulated solutions and (**b**) the two natural waters. Note the logarithmic concentration axis and the far lower release in the natural waters (≤0.087 mg/L versus 0.16–1.43 mg/L in the simulated matrices). Error bars show the standard deviation of triplicate filtrate samples. Different lowercase letters indicate significant differences after Benjamini–Hochberg FDR correction (adjusted *p* < 0.05).

CuO nanoparticles dissolved slowly in the simulated matrices, releasing 0.16–1.43 mg/L Cu^2+^ over 72 h with the highest release in the NOM solution, where copper complexation by the organic ligand promoted dissolution (Figure 3a). In the natural waters the release was markedly suppressed—dissolved Cu^2+^ never exceeded 0.026 mg/L in Taihu Lake water, 55-fold below the corresponding NOM-solution value—and reached only 0.087 mg/L in Suzhou River water at 72 h, corresponding to at most 0.11% of the nominal dose (Figure 3b, Table 5). The fitted rate constants reflect the same pattern, with k = 0.072 h^−1^ for the late rising phase in Suzhou River water but only 0.004 h^−1^ in Taihu Lake water.

**Figure 4:**
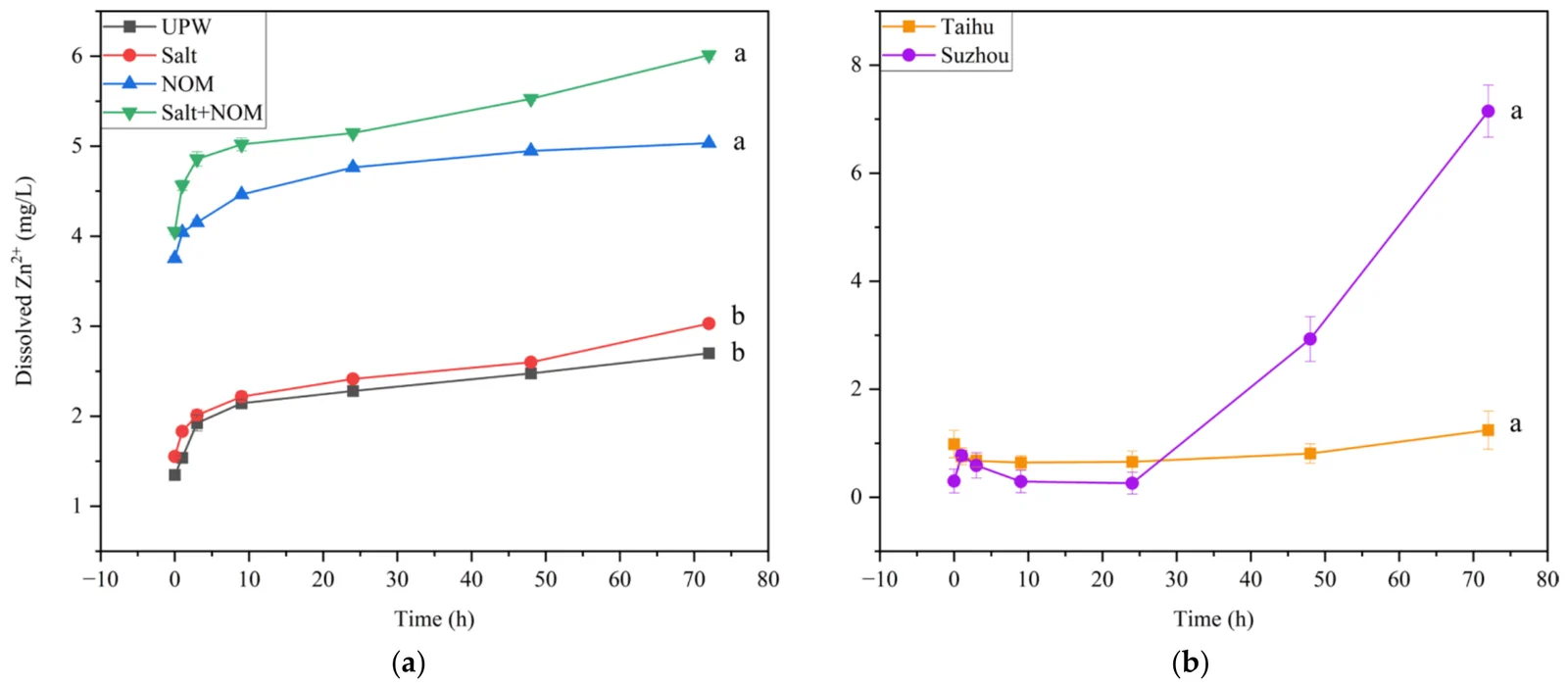
Dissolved Zn^2+^ released from ZnO nanoparticles (100 mg/L) over 72 h in (**a**) the four simulated solutions and (**b**) the two natural waters. In Suzhou River water the concentration surged from 0.26 to 7.15 mg/L between 24 and 72 h. Error bars show the standard deviation of triplicate filtrate samples. Different lowercase letters indicate significant differences after Benjamini–Hochberg FDR correction (adjusted *p* < 0.05).

ZnO nanoparticles dissolved much more readily [30,31]. In the simulated matrices the released Zn^2+^ increased continuously to 2.70–6.01 mg/L, with NOM-containing solutions again promoting release (Figure 4a). The two natural waters diverged sharply: Taihu Lake water sustained a quasi-steady dissolved concentration of 0.64–1.24 mg/L Zn^2+^ across the whole 72 h, whereas Suzhou River water showed a delayed surge in which the concentration jumped from 0.26 mg/L at 24 h to 2.93 mg/L at 48 h and 7.15 mg/L at 72 h (Figure 4b), a 24-fold increase that released 8.9% of the nominal ZnO dose (Table 4).

**Figure 5:**
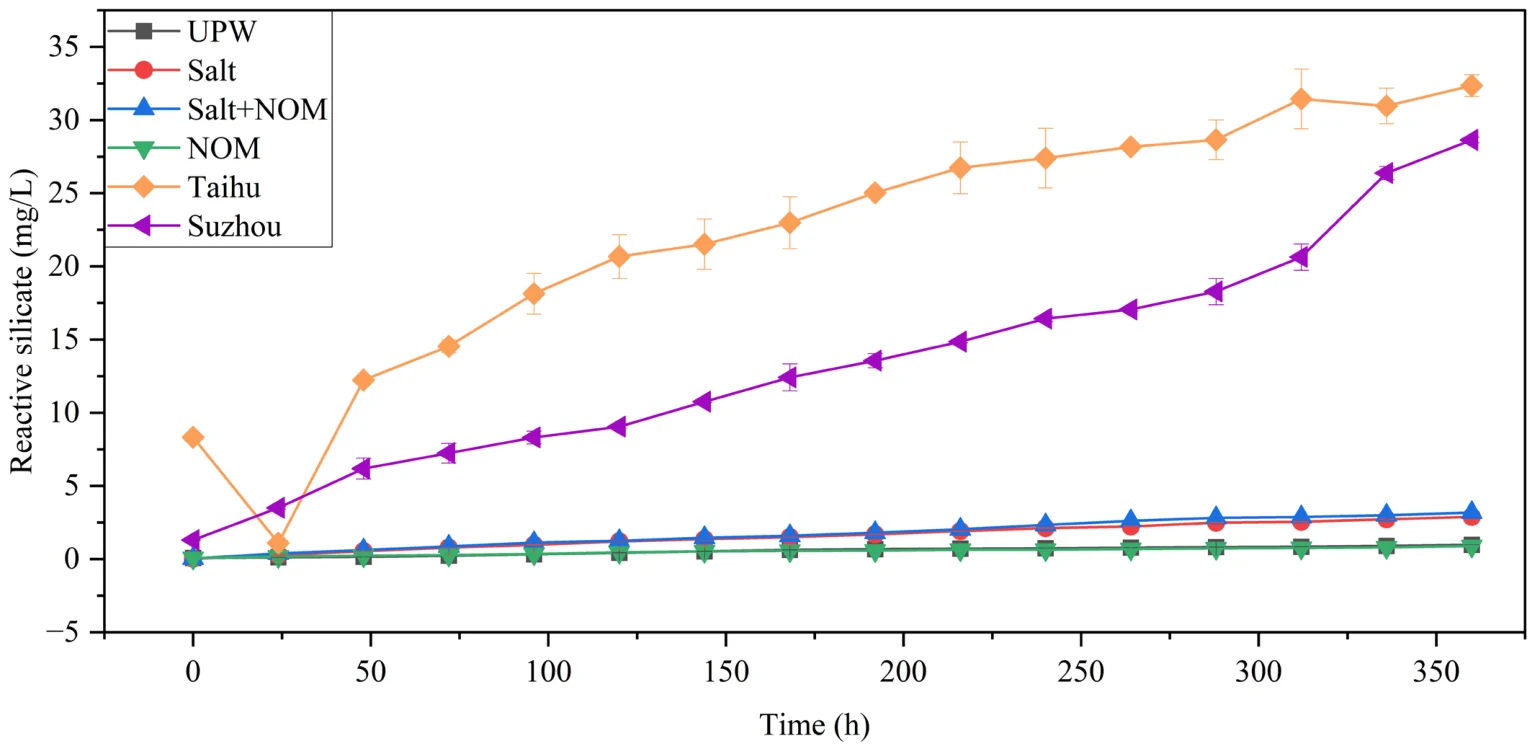
Reactive silicate in SiO_2_ nanoparticle suspensions (100 mg/L) over 360 h in the six test solutions. The t = 0 values of the natural waters reflect their background silicate content; the subsequent rise reflects nanoparticle dissolution. Error bars show the standard deviation of triplicate filtrate samples.

The SiO_2_ suspensions released reactive silicate continuously over the 15 d experiment (Figure 5). In the simulated matrices the final concentrations reached only 0.90–3.17 mg/L (0.9–3.2% of the dose), but in the natural waters the silicate concentration rose from background levels of 8.33 and 1.30 mg/L to 32.36 and 28.64 mg/L, respectively, corresponding to net releases of 24.0% and 27.3% of the nominal dose over 15 d (Table 4). The faster dissolution in the natural waters is consistent with their higher pH (7.2–7.8 versus 5.5–6.3 in the simulated matrices).

### 3.3. Acute Toxicity to Vibrio fischeri

**Figure 6:**
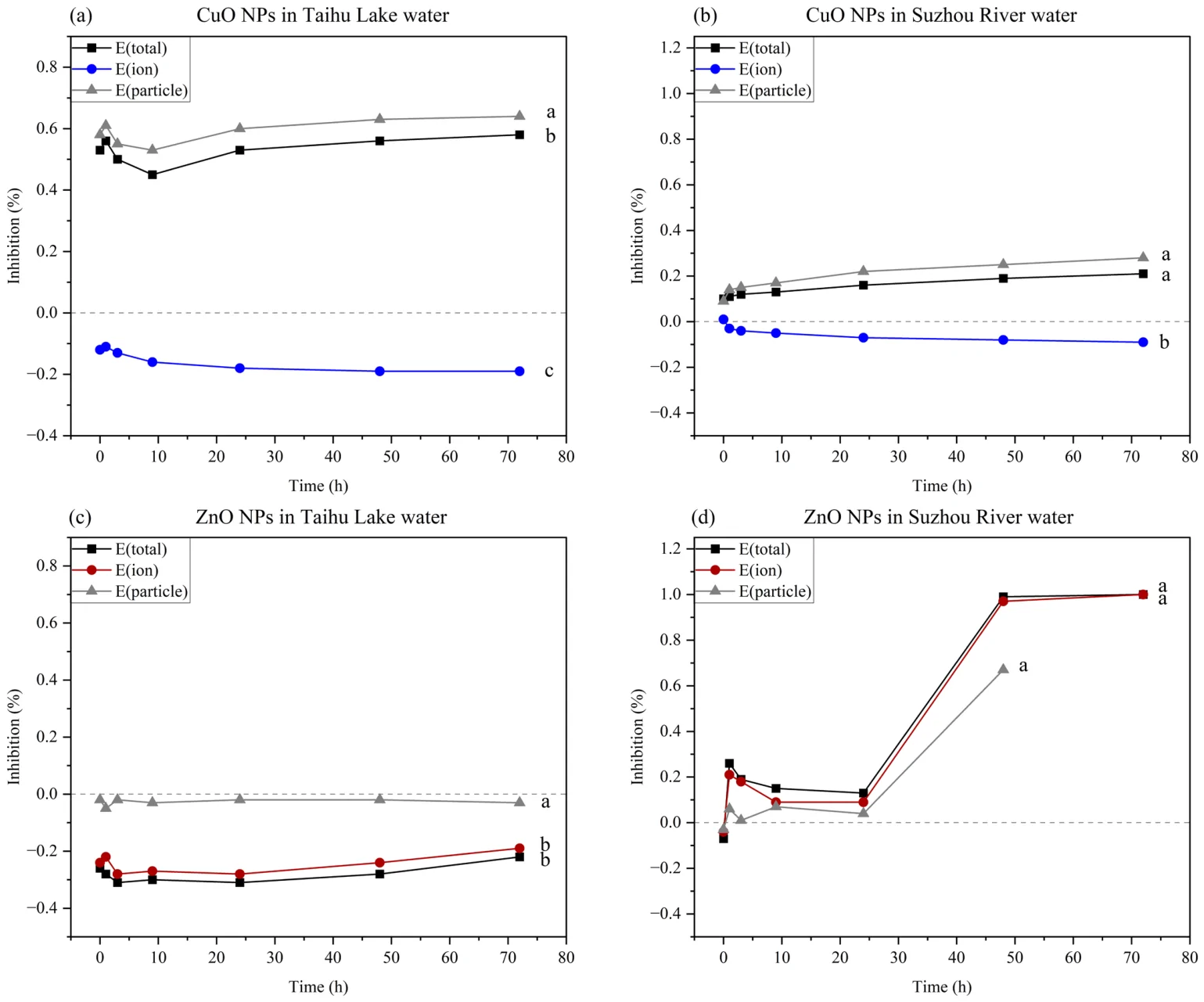
Time course of the inhibition of *V. fischeri* luminescence by CuO and ZnO nanoparticle suspensions (100 mg/L) in the two natural waters: (**a**) CuO in Taihu Lake water, (**b**) CuO in Suzhou River water, (**c**) ZnO in Taihu Lake water, (**d**) ZnO in Suzhou River water. E(total), whole suspension; E(ion), dissolved fraction (0.02 µm filtrate); E(particle), particle-attributed component obtained as 1 − (1 − E(total))/(1 − E(ion)) following Equation (2). Positive values indicate inhibition and negative values hormesis (stimulation of luminescence). Data are means of triplicate wells. Different lowercase letters indicate significant differences among treatments (adjusted *p* < 0.05).

The CuO suspensions inhibited luminescence at every sampling time in both natural waters, with E(total) between 0.45% and 0.58% in Taihu Lake water and between 0.10% and 0.21% in Suzhou River water, where the inhibition grew slowly with time (Figure 6a,b). The dissolved fractions produced the opposite signal: E(ion) was negative at all Taihu time points (−0.11% to −0.19%) and at all but the first Suzhou time point, indicating weak stimulation of luminescence by the released Cu^2+^ at these low concentrations. The particle-attributed component E(particle) therefore carried the entire inhibitory effect, at 0.53–0.64% in Taihu Lake water and 0.09–0.28% in Suzhou River water.

ZnO behaved differently in the two waters. In Taihu Lake water both the suspension and the dissolved fraction stimulated luminescence throughout the 72 h (E(total) −0.22% to −0.31%; E(ion) −0.19% to −0.28%), and E(particle) remained between −0.02% and −0.05% (Figure 6c). In Suzhou River water the response reversed over time: E(total) started at −0.07% (stimulation), became slightly positive during 1–24 h, and then rose to 0.99% and 1.00% at 48 and 72 h, tracking the surge of dissolved Zn^2+^ almost exactly, with E(ion) reaching 0.97% and 1.00% and E(particle) staying no higher than 0.07% in the first 24 h (Figure 6d).

**Figure 7:**
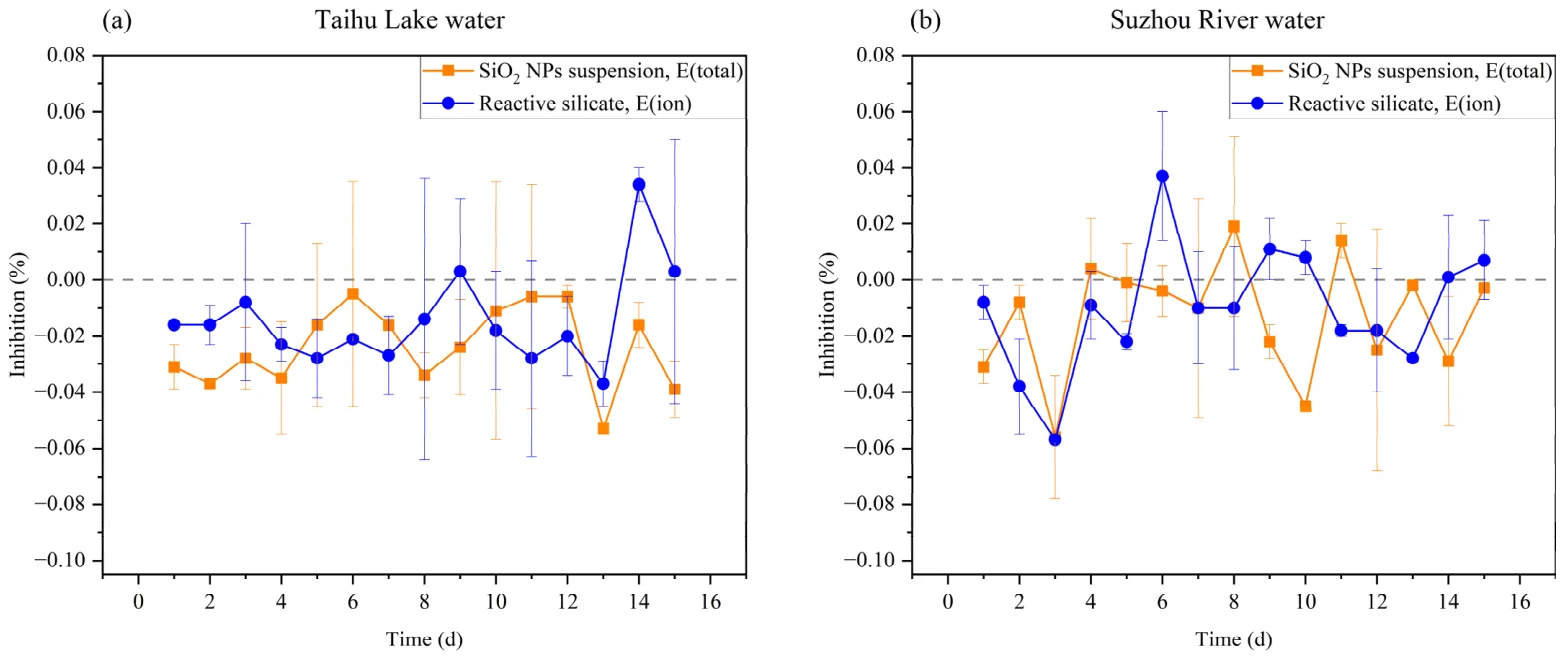
Daily inhibition of *V. fischeri* luminescence over 15 d by SiO_2_ nanoparticle suspensions (100 mg/L, E(total)) and by the dissolved silicate fraction (E(ion)) in (**a**) Taihu Lake water and (**b**) Suzhou River water. All daily means remain within −0.06% to 0.04%, indicating the absence of acute toxicity and a weak hormetic tendency. Error bars show the standard deviation of triplicate wells.

SiO_2_ nanoparticles produced no luminescence inhibition in either water over 15 d. The daily means of the suspension assays ranged from −0.053% to −0.005% in Taihu Lake water and from −0.056% to 0.019% in Suzhou River water (Figure 7), with 15 d averages of −0.024% and −0.013% that were both significantly below zero in one-sample *t*-tests (t = −6.42, *p* < 0.001; t = −2.44, *p* = 0.029), indicating a mild but consistent stimulation of luminescence (hormesis). The silicate filtrates showed the same weak hormetic tendency (means −0.014% and −0.010%), and neither the suspensions nor the filtrates displayed any temporal correlation with the rising silicate concentration (r ≤ 0.30, *p* ≥ 0.27). Marginal contribution partitioning was omitted for SiO_2_ systems since both E(total) and E(ion) fell within the experimental noise of the *V. fischeri* assay.

### 3.4. Toxicity-Source Apportionment

**Figure 8:**
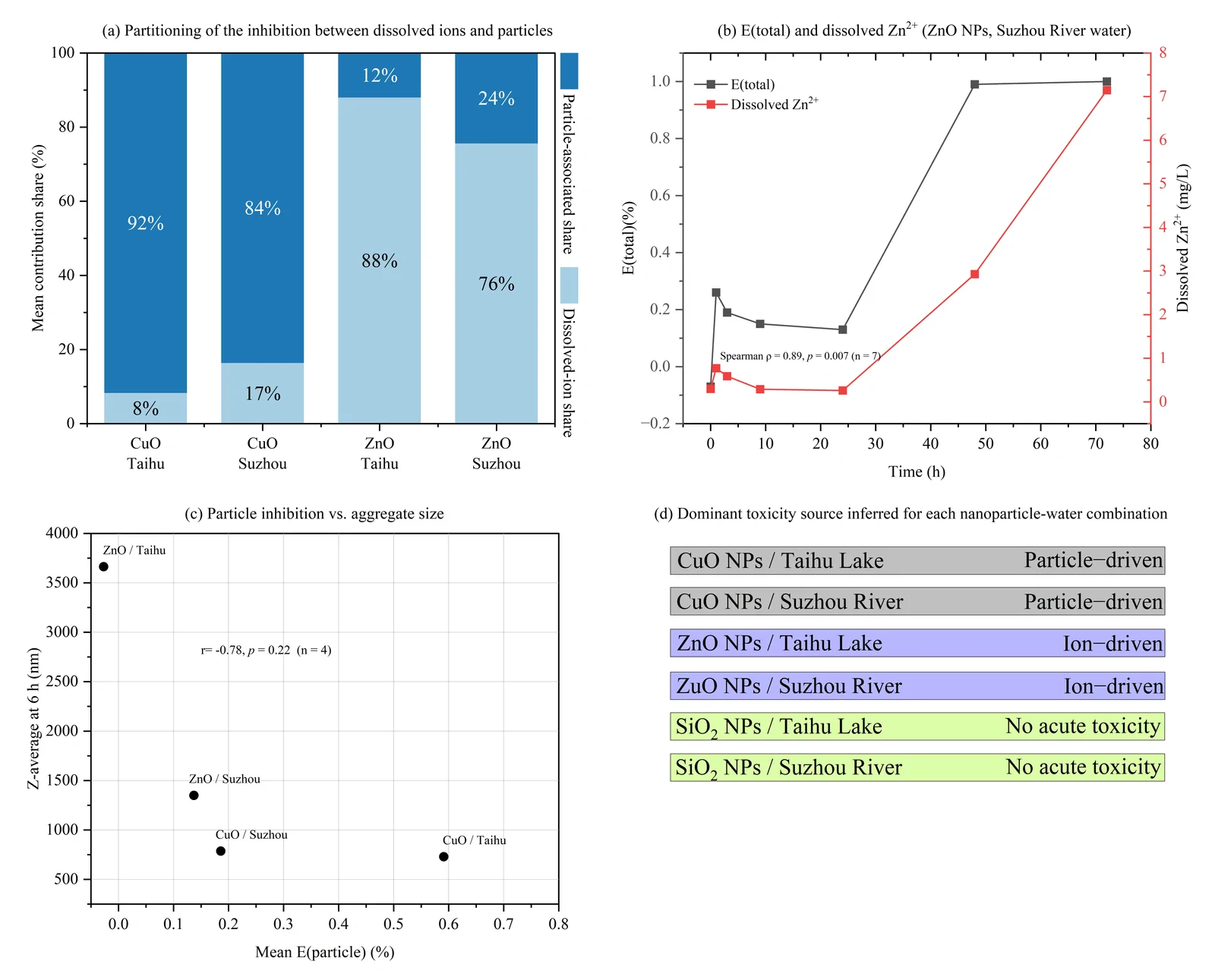
Toxicity-source apportionment. (**a**) Stacked contribution shares of the ion-attributed E(ion) and particle-attributed E(particle) components to the measured |E(total)| in the four CuO/ZnO systems. (**b**) Coupled rise in E(total) and dissolved Zn^2+^ for ZnO nanoparticles in Suzhou River water (Spearman ρ = 0.89, *p* = 0.007, n = 7). (**c**) Time-averaged E(particle) of the four CuO/ZnO systems plotted against the Z-average hydrodynamic diameter at 6 h; the correlation is not significant (r = −0.78, *p* = 0.22, n = 4). (**d**) Dominant toxicity source inferred for each nanoparticle–water combination.

Partitioning the measured effects according to Equation (2) yields a clean separation between the two transition-metal oxides (Figure 8, Table 6). In both CuO systems the particle-attributed component dominated at all seven time points, with mean shares of 91.6% in Taihu Lake water and 83.5% in Suzhou River water, and the paired *t*-tests confirmed that E(total) differed strongly from E(ion) (t = 29.78 and 6.94, both *p* < 0.001). In the ZnO systems the paired difference was significant in Taihu Lake water (t = −7.13, *p* < 0.001) but not in Suzhou River water (t = 1.81, *p* = 0.12), where the late-time responses of the suspension and the dissolved fraction converged. The dissolved-ion shares were correspondingly small (8.4% and 16.5%). In the two ZnO systems the pattern reversed: the ion-attributed component was the larger of the two at every time point, with mean shares of 88.1% (Taihu Lake water) and 75.7% (Suzhou River water). The association between E(total) and the dissolved-ion concentration was positive within all four systems (Pearson r = 0.72–0.98; Table 6), and for ZnO in Suzhou River water the coupled rise in the two series was essentially synchronous (Spearman ρ = 0.89, *p* = 0.007; Figure 8b).

Across the four CuO/ZnO systems, the time-averaged particle-attributed component showed no simple dependence on the 6 h aggregate size (Figure 8c; r = −0.78, *p* = 0.22, n = 4), which indicates that particle toxicity was not merely proportional to aggregate dimensions [32,33]. The overall classification is summarized in Figure 8d: the acute effect of CuO nanoparticles in both natural waters is particle-driven, the acute effect of ZnO nanoparticles is ion-driven, and SiO_2_ nanoparticles exert no acute toxicity in either water.

## 4. Discussion

### 4.1. CuO Nanoparticles: A Particle-Driven Acute Effect

The decomposition shows that the persistent inhibition by CuO suspensions in both natural waters cannot be explained by dissolved copper, which stayed below 0.09 mg/L and, when assayed alone, mildly stimulated the bacteria. The particle-attributed component, obtained as the difference 1 − (1 − E(ion))(1 − E(total)), carried 81.1–94.0% of the effect and was positive at every time point. This particle-specific action is consistent with the comparative literature: toxicity of CuO nanoparticles differing from that of dissolved copper has been demonstrated in *D. melanogaster* [16], in *T. tubifex* [15], in microalgae challenged with both forms [34], and in freshwater mesocosms where particle fate governed the observed toxicity [17]. Mechanistically, contact-dependent damage such as membrane disruption and locally elevated reactive-oxygen-species production at the particle surface offers a plausible route for the bacterial endpoint, and in vertebrate models CuO nanoparticles additionally produce acute lethality in the freshwater fish *Labeo rohita* [35], reproductive impairment in zebrafish [36], and, in recent work, cuproptosis-like mitochondrial dysfunction [37], suggesting that the particle-specific hazard generalizes beyond bacteria.

Two observations about the dissolved fraction deserve emphasis. First, the strong suppression of CuO dissolution in the natural waters relative to the simulated matrices, likely reflecting phosphate/carbonate precipitation of released copper and surface passivation by natural ligands, means that in real water bodies the ion channel remains minor even though NOM alone promotes dissolution in simplified media [12]. Ligand-driven dissolution of CuO has also been shown for anthropogenic chelators such as glyphosate [38], so waters heavily loaded with synthetic ligands could shift this balance. Second, the negative E(ion) values show that the released Cu^2+^ at these sub-µg/L to 0.09 mg/L levels acted as a mild stimulant of bacterial luminescence, a response consistent with the widespread hormetic behavior of microorganisms toward low metal doses [39] and with the specific caveat that such biphasic responses bias luminescent-bacterial test results [27]. The implication for interpretation is direct: any assay that reports only the whole-suspension signal would underestimate the particle hazard because the stimulatory ion component partially masks it, and the water-chemistry dependence of copper bioavailability documented for Daphnia across hardness gradients [40] implies that the same masking would vary between waters.

### 4.2. ZnO Nanoparticles: A Dissolution-Driven Acute Effect

For ZnO the decomposition points unambiguously to the dissolved fraction. The ion-attributed component contributed 56.8–95.6% of the measured effect in both waters and was the larger component at every time point, in line with the mechanism established for *V. fischeri*, where ZnO nanoparticle toxicity was attributed to dissolved Zn^2+^ [18], and with rotifer data in which the ion fraction recapitulated the nanoparticle response [19]. The Suzhou River series is the most instructive: the suspension was stimulatory or near-neutral while dissolution lagged, and inhibition appeared exactly as the dissolved concentration crossed from 0.26 mg/L toward 2.9–7.1 mg/L between 24 and 72 h, with the two series rising in near synchrony. A delayed dissolution burst of this kind has been reported for ZnO in surface waters, where dissolution kinetics at low loadings are governed by slow surface-ligand exchange rather than by equilibrium solubility alone [13], and exogenous factors such as irradiation and co-occurring particles can further accelerate ion release [41]. The Taihu Lake series stayed within the stimulatory window because dissolved Zn^2+^ remained near 1 mg/L, below the apparent threshold that separates hormesis from inhibition for this bacterium, and this biphasic pattern accords with mechanistic reviews of ZnO toxicity in freshwater environments [42]; the strongly aggregated state of ZnO in that water (3.4–3.7 µm, Figure 1) may have retarded dissolution by reducing the reactive surface area, a possibility consistent with the organic-matter-dependent aggregation behavior documented for ZnO [43] and with its kinetic control in simulated freshwater media [44]. Because zinc bioavailability to freshwater organisms is tightly controlled by dissolved organic carbon, hardness, and pH through speciation [45], the ion-dominated hazard identified here should be re-evaluated water-by-water in monitoring programs.

### 4.3. SiO_2_ Nanoparticles: No Acute Toxicity Despite Substantial Dissolution

SiO_2_ suspensions and their silicate filtrates produced no acute inhibition in either natural water across 15 d, even though dissolved 32.6% in Taihu Lake water and 28.6% in Suzhou River water of the nominal dose. The absence of an acute effect is consistent with the evidence that manufactured silica nanoparticles show weak or no acute toxicity in most aquatic test systems [21,22], with low-level effects appearing mainly under chronic or high-dose conditions in fish early life stages [46] and in freshwater bivalves [47], and with the low response of bioluminescent bacteria to SiO_2_ abrasives [24]. The persistent weak stimulation of luminescence (daily means predominantly negative) mirrors the hormesis documented for nanoparticles in microalgae, where low doses upregulate growth-promoting pathways [48], and it reinforces the point that luminescent-bacterial endpoints must treat stimulation as a real biological response rather than experimental noise [27]. The lack of any correlation between inhibition and the rising silicate concentration further confirms that the dissolved fraction was not merely non-toxic but effectively inert at the levels reached, so that neither component of the SiO_2_ system requires acute-effect management in the two waters studied.

### 4.4. Implications for Toxicity-Source Management

The clean material-dependent separation of toxicity sources carries practical consequences. For CuO nanoparticles, measures that reduce particle transport and contact—sedimentation basins, enhanced coagulation, and interception of suspended solids—should control most of the acute effect, whereas reducing ion release matters comparatively little in waters of the type studied here. For ZnO nanoparticles the priority inverts: the acute effect follows the dissolved zinc, so treatments that slow dissolution (e.g., surface coatings or pH/ligand management) and models of dissolved-zinc bioavailability should drive risk management, and the form-specific probabilistic risk assessment of nano-ZnO in freshwaters, which weights the dissolved and particulate forms explicitly, offers a ready template [49]. Mixtures should not be forgotten, because co-occurring Cu and ZnO nanoparticles interact through their shared metal chemistry, with NOM modulating their joint toxicity and accumulation in daphnids [50], and because chronic exposures of fish to these oxides produce oxidative stress, genotoxicity, and histopathological change [51,52]. Finally, although dissolved Cu^2+^ and the SiO_2_ system showed no acute effect in this study, both represent persistent, low-reactivity forms that can be taken up by primary consumers and transferred along food chains: nano-TiO_2_, for example, bioaccumulates and biomagnifies in aquatic food chains [53], and meta-analyses show that nondissolvable engineered nanomaterials accumulate in freshwater organisms at levels relevant to regulatory assessment [54]. Management of the dominant toxicity source should therefore proceed in parallel with surveillance of the secondary sources, because substances that are not acutely toxic can still generate chronic risk through trophic transfer. The present conclusions derive from a single bacterial species, a single pulse dose, and short exposure windows, so extrapolation to other trophic levels and to chronic regimes remains a priority for future work.

One methodological limitation should also be noted: we did not perform time-series incubations of nanoparticle-free natural-water blanks to track potential intrinsic luminescence-signal drift of the raw water matrices over stirring-incubation. Internal plate-level controls were applied for each measurement, but parallel incubated matrix blanks are recommended in future time-resolved nanotoxicity assays to fully rule out time-dependent baseline drift originating from natural-water constituents.

In addition, further studies employing agar-well or disc-diffusion approaches, together with measurements of minimum inhibitory concentration (MIC) and minimum bactericidal concentration (MBC), would provide complementary insights into the antibacterial activity of these metal-oxide nanoparticles on solid media. Such follow-up work could help compare nanoparticle-induced effects between short-term liquid-phase acute luminescence assays and long-term solid-culture inhibition endpoints.

## 5. Conclusions

Parallel measurements in Taihu Lake water and Suzhou River water show that the acute toxicity source of nano-metal oxides is material-specific within the same water matrices. CuO nanoparticles inhibited *V. fischeri* luminescence through their particle component, which contributed 81.1–94.0% of the effect while the released Cu^2+^ acted as a mild stimulant at the low concentrations sustained in natural water; the dissolved fraction of ZnO nanoparticles, by contrast, contributed 56.8–95.6% of the effect, with inhibition in Suzhou River water emerging in synchrony with a dissolution surge that reached 7.15 mg/L Zn^2+^, whereas in Taihu Lake water the system remained within its stimulatory window. SiO_2_ nanoparticles and the released reactive silicate produced no acute toxicity over 15 d despite a 24–27% net dissolution of the nominal dose, showing instead a weak hormetic tendency. These results imply material-directed risk management—particle-transport control for CuO, dissolution suppression for ZnO—and, because the non-acutely toxic fractions (dissolved Cu^2+^, silicate, and persistent particles) can still bioaccumulate and move along food chains, monitoring should track these secondary sources alongside the dominant ones.

## Figures and Tables

**Table 1:** Characteristics of CuO, ZnO and SiO, NPs.

Nanoparticle	Initial Size (nm)	Morphology	Purity	Surface Coating	Subsequent Treatment
CuO NPs	45.1 ± 8.6 (n = 36)	Sphere	>99%	None	None
ZnO NPs	56.9 ± 7.1 (n = 40)	Sphere (a small amount of rod-like)	>97%	None	None
SiO_2_ NPs	53.3 ± 4.7 (n = 30)	Sphere	99.50%	None	None

**Table 2:** Physicochemical properties of the Taihu Lake and Suzhou River water samples (after 0.45 µm filtration). ND: not detected.

Property (Unit)	Taihu Lake Water	Suzhou River Water
Water temperature (°C)	21	17
pH	7.80	7.28
Conductivity (µS/cm)	39.7	79.0
Total dissolved solids (mg/L)	19.8	39.5
Total organic carbon (mg/L)	10.06	15.48
Ionic strength (mmol/L)	3.2	3.4
K^+^ (mg/L)	4.82	10.34
Ca^2+^ (mg/L)	6.42	11.27
Na^+^ (mg/L)	20.94	34.58
Mg^2+^ (mg/L)	8.44	10.85
Al^3+^ (mg/L)	<0.05	<0.05
Cu^2+^ (mg/L)	ND	ND
Zn^2+^ (mg/L)	ND	ND
Cl^−^ (mg/L)	21.43	37.92
NO_3_^−^ (mg/L)	5.15	67.78
SO_4_^2−^ (mg/L)	63.87	<0.08
PO_4_^3−^ (mg/L)	<0.10	<0.10
HCO_3_^−^ (mg/L)	1.25	ND

**Table 3:** Reagents used for reactive silicate determination by the silicomolybdenum blue method.

Reagent	Concentration	Note
HCl	1:1 (*v*/*v*)	—
(NH_4_)_6_Mo_7_O_24_·4H_2_O	100 g/L	pH adjusted to 7.5 ± 0.5 with ammonia
H_2_C_2_O_4_·2H_2_O	75 g/L	—
SiO_2_ standard solution	0.01 g/L	prepared by 10-fold dilution of the 100 mg/L stock
Reducing agent (Na_2_SO_3_:NaHSO_3_:1,2,4-aminonaphtholsulfonic acid)	5:150:2.5 (g/L)	filtered, stored cold in the dark

**Table 4:** Composition of the solid and liquid culture media used for *V. fischeri* (pH 7.0 ± 0.2, made up to 1 L with UPW).

Component	Concentration (g/L)	Component	Concentration (g/L)
NaCl	30	MgSO_4_·7H_2_O	0.20
NaH_2_PO_4_·2H_2_O	6.90	Peptone	5.00
K_2_HPO_4_·3H_2_O	2.75	Yeast extract	0.50
(NH_4_)_2_HPO_4_	0.50	Agar (solid medium only)	12.0
—	—	Glycerol	3 mL/L

**Table 5:** Dissolution kinetics of the three nanoparticles in the six test solutions. C_0_ and C_enk_ denote the dissolved-ion concentration at the start and end of the experiment (72 h for CuO and ZnO, 360 h for SiO_2_); k is the pseudo-first-order rate constant fitted to the rising phase; % is the fraction of the 100 mg/L nominal dose released at the end (for SiO_2_ in the natural waters the value is the net release after subtraction of the background silicate measured at t = 0).

Solution	C_0_ (mg/L)	C_enk_ (mg/L)	k (h^−1^)	R^2^	Released (%)
CuO nanoparticles (dissolved Cu ^2+^, 72 h)					
UPW	0.016	0.155	0.0126	0.154	0.19
Inorganic salt solution	0.477	0.530	0.0012	0.422	0.66
NOM solution	0.735	1.427	0.0070	0.612	1.79
Salt + NOM solution	0.659	1.176	0.0056	0.467	1.47
Taihu Lake water	0.020	0.026	0.0041	0.828	0.03
Suzhou River water	0.000	0.087	0.0721	0.948	0.11
ZnO nanoparticles (dissolved Zn ^2+^, 72 h)					
UPW	1.348	2.700	0.0075	0.682	3.36
Inorganic salt solution	1.553	3.030	0.0073	0.810	3.77
NOM solution	3.753	5.034	0.0035	0.757	6.27
Salt + NOM solution	4.052	6.013	0.0041	0.787	7.48
Taihu Lake water	0.986	1.241	0.0053	0.364	1.54
Suzhou River water	0.298	7.147	0.0394	0.730	8.90
SiO _2_ nanoparticles (reactive silicate, 360 h)					
UPW	0.075	0.979	0.0064	0.838	0.98
Inorganic salt solution	0.032	2.892	0.0082	0.673	2.89
NOM solution	0.070	0.899	0.0053	0.794	0.90
Salt + NOM solution	0.035	3.172	0.0081	0.668	3.17
Taihu Lake water	8.327	32.356	0.0052	0.510	24.0 (net)
Suzhou River water	1.303	28.643	0.0063	0.842	27.3 (net)

**Table 6:** Toxicity-source summary for the six nanoparticle–water systems. E(total), E(ion), and E(particle) denote the inhibition of *V. fischeri* luminescence at each sampling time point (positive: inhibition; negative: hormesis). E(particle) is back-calculated from E(total) =1 − (1 − E(ion))(1 − E(particle)). Ion and particle shares represent the per-time-point marginal-contribution proportions derived from Equations (3) and (4). These calculations are not performed for SiO_2_ because both components fell within the experimental noise of the assay. r is the Pearson correlation between E(total) and the dissolved-ion concentration over the seven sampling times (n = 7; for SiO_2_, over 15 daily samples with the reactive silicate concentration). Reported *p*-values are Benjamini–Hochberg FDR-adjusted *p*-values accounting for multiple testing.

System	Time (h)	C(Ion) (mg/L)	E(Total) (%)	E (Ion) (%)	E (Particle) (%)	Ion Share (%)	Particle Share (%)	Larger Component	r (*p*)	Classification
CuO in Taihu Lake water	0	0.020	0.53	−0.12	0.58	7.2	92.8	particle (7/7 time points)	0.718 (0.069)	Particle-driven
	1	0.021	0.56	−0.11	0.61	6.0	94.0			
	3	0.018	0.5	−0.13	0.55	8.6	91.4			
	9	0.020	0.45	−0.16	0.53	10.9	89.1			
	24	0.021	0.53	−0.18	0.60	9.2	90.8			
	48	0.025	0.56	−0.19	0.63	8.6	91.4			
	72	0.026	0.58	−0.19	0.64	8.2	91.8			
CuO in Suzhou River water	0	0.000	0.10	0.01	0.09	9.3	90.7	particle (7/7 time points)	0.895 (0.006)	Particle-driven
	1	0.000	0.11	−0.03	0.14	15.2	84.8			
	3	0.001	0.12	−0.04	0.15	17.9	82.1			
	9	0.002	0.13	−0.05	0.17	18.9	81.1			
	24	0.003	0.16	−0.07	0.22	18.8	81.2			
	48	0.048	0.19	−0.08	0.25	18.2	81.8			
	72	0.087	0.21	−0.09	0.28	17.5	82.5			
ZnO in Taihu Lake water	0	0.986	−0.26	−0.24	−0.02	90.8	9.2	ion (7/7 time points)	0.980 (<0.001)	Ion-driven (hormetic)
	1	0.758	−0.28	−0.22	−0.05	79.1	20.9			
	3	0.673	−0.31	−0.28	−0.02	91.8	8.2			
	9	0.641	−0.30	−0.27	−0.03	88.0	12.1			
	24	0.657	−0.31	−0.28	−0.02	91.8	8.2			
	48	0.809	−0.28	−0.24	−0.02	90.8	9.2			
	72	1.241	−0.22	−0.19	−0.03	84.6	15.4			
ZnO in Suzhou River water	0	0.298	−0.07	−0.04	−0.03	56.9	43.1	ion (6/6 time points)	0.867 (0.011)	Ion-driven
	1	0.772	0.26	0.21	0.06	80.6	19.4			
	3	0.589	0.19	0.18	0.01	95.6	4.4			
	9	0.291	0.15	0.09	0.07	56.8	43.2			
	24	0.260	0.13	0.09	0.04	70.4	29.6			
	48	2.929	0.99	0.97	0.67	94.1	5.9			
	72	7.147	1	1	—	—	—			
SiO_2_ in Taihu Lake water	24	10.96	−0.031	−0.016	—	—	—	—	0.124 (0.660)	No acute toxicity
	48	12.23	−0.037	−0.016	—	—	—			
	72	14.54	−0.028	−0.008	—	—	—			
	96	18.13	−0.035	−0.023	—	—	—			
	120	20.67	−0.016	−0.028	—	—	—			
	144	21.52	−0.005	−0.021	—	—	—			
	168	22.98	−0.016	−0.027	—	—	—			
	192	25.02	−0.034	−0.014	—	—	—			
	216	26.73	−0.024	0.003	—	—	—			
	240	27.40	−0.011	−0.018	—	—	—			
	264	28.17	−0.006	−0.028	—	—	—			
	288	28.65	−0.006	−0.02	—	—	—			
	312	31.44	−0.053	−0.037	—	—	—			
	336	30.96	−0.016	0.034	—	—	—			
	360	32.36	−0.039	0.003	—	—	—			
SiO_2_ in Suzhou River water	24	3.50	−0.031	−0.008	—	—	—	—	0.096 (0.735)	No acute toxicity
	48	6.19	−0.008	−0.038	—	—	—			
	72	7.24	−0.056	−0.057	—	—	—			
	96	8.31	0.004	−0.009	—	—	—			
	120	9.05	−0.001	−0.022	—	—	—			
	144	10.75	−0.004	0.037	—	—	—			
	168	12.42	−0.01	−0.01	—	—	—			
	192	13.56	0.019	−0.01	—	—	—			
	216	14.86	−0.022	0.011	—	—	—			
	240	16.43	−0.045	0.008	—	—	—			
	264	17.05	0.014	−0.018	—	—	—			
	288	18.28	−0.025	−0.018	—	—	—			
	312	20.63	−0.002	−0.028	—	—	—			
	336	26.38	−0.029	0.001	—	—	—			
	360	28.64	−0.003	0.007	—	—	—			

## Data Availability

Data will be made available on request.

## Abbreviations

NPs	Nanoparticles
NOM	Natural organic matter
UPW	Ultrapure water
*V. fischeri*	*Vibrio fischeri*
*T. tubifex*	*Tubifex tubifex*
*D. melanogaster*	*Drosophila melanogaster*
TEM	Transmission electron microscopy
